# Studies of the potential of a native natural biosorbent for the elimination of an anionic textile dye Cibacron Blue in aqueous solution

**DOI:** 10.1038/s41598-021-88657-y

**Published:** 2021-05-06

**Authors:** Hocine Grabi, Fazia Derridj, Wahiba Lemlikchi, Erwann Guénin

**Affiliations:** 1 Laboratory of Applied Chemistry and Chemical Enginering, Faculty of Sciences, UMMTO, 15000 Tizi Ouzou, Algeria; 2 Laboratory of Physics and Chemistry of Materials, Faculty of Sciences, UMMTO, 15000 Tizi Ouzou, Algeria; 3 University Algiers 1-Benyoucef Benkhedda, 02 Street Didouche Mourad, 16000 Algiers, Algeria; 4 Laboratory of Integrated Transformations of Renewable Matter, University of Technology of Compiègne, 60200 Compiègne, France

**Keywords:** Environmental sciences, Chemistry, Materials science

## Abstract

This work is devoted to the adsorption of Cibacron Blue (CB) an anionic textile dye, on bean peel (BP) an agricultural waste with neither activation nor carbonization. The adsorption was realized in batch configuration at ambient temperature in acidic medium. The adsorbent was characterized by FTIR, SEM and BET analyses; the equilibrium isotherms and kinetics were also studied. It has been found that this waste could be used as a low-cost biosorbent for CB elimination under optimal working conditions. The rate of CB elimination reaches 95% on bean bark (3.6 g/L) at pH 2.2 and a reject concentration of 25 mg/L. The pseudo-second-order describes suitably the experimental data and the external diffusion is the rate-determining step. The Freundlich isotherm fits better the CB adsorption with a correlation coefficient (R^2^) of 0.94 and an RMSE = 1.5115. The negative enthalpy (ΔH) and free enthalpy (ΔG°) indicate a physical and spontaneous nature of the CB biosorption onto the biomaterial.

## Introduction

During the past decades, public environmental concerns have pushed the chemical industry to reconsider its business strategies in terms of impact on the environment and sustainability. Thus, environmental protection is a relevant preoccupation for scientists. Indeed, several industrial activities continue to produce various pollutants, including organic molecules, toxic metals, dyes and pesticides, which generate nuisances and represents a serious threat to for the aquatic environment, particularly in developing countries^1^. The pollution of the phreatic sources prompted scientist to develop novel strategies for the preservation of our planet. Currently, water pollution remains one of the most serious environmental problems in the world^2^.

The textile industry contains hundreds of dyes of synthetic origin and some of them are highly carcinogenic. Additionally, they are difficult to biodegrade^3^^,^^4^. They also weaken considerably the penetration of the solar light in water, affecting thereby the photosynthesis^5–7^. To limit the impact of hazardous dyes, many physicochemical treatments, are employed such as flocculation/coagulation^8^, ion exchange^9^, photocatalysis^10^. The list may be extented to advanced oxidation processes^11^, Membrane processes^12^, Fenton oxidation^13^ and biological treatment. However treatments often become inefficient for dye elimination at low concentrations^14^.

In this regard, the adsorption is an inexpensive alternative and simple to implement. During the last decades, natural materials like olive kernels^15^; date kernels^16^, orange peel^17^, potato peel^18^, pomegranate peel^19^, apricot shell^20^, banana peel^21^, bagasse agave^22^, cocoa pods^23^, avocado skin^24^, sugarcane bagasse^25^ have found application in the water decontamination. Components based on forestry and agricultural wastes, including cellulose, hemicellulose of tannins, lignin, pectin, simple sugars and water; contain various functional groups with a potential adsorption for various organic and inorganic pollutants^26^.

This contribution is devoted to the evaluation of the capacity of a biomaterial "bean peel", derived from plant waste, available in Kabylia (Algeria), for the elimination of Cibacron Blue (CB) an anionic dye used in the textile industry. Statistically, the production of "bean peel" amount to about 43 million tons. Therefore, bean bark is economically beneficial at large scale.

The study was carried out with the aim of optimizing the parameters influencing the biosorption related to intrinsic and extrinsic factors^27^. Moreover, the modeling of the different kinetic models for example the pseudo-first-order and pseudo second order^28,29^ was performed.

## Materials and methods

### Adsorbent

**Figure 1:**
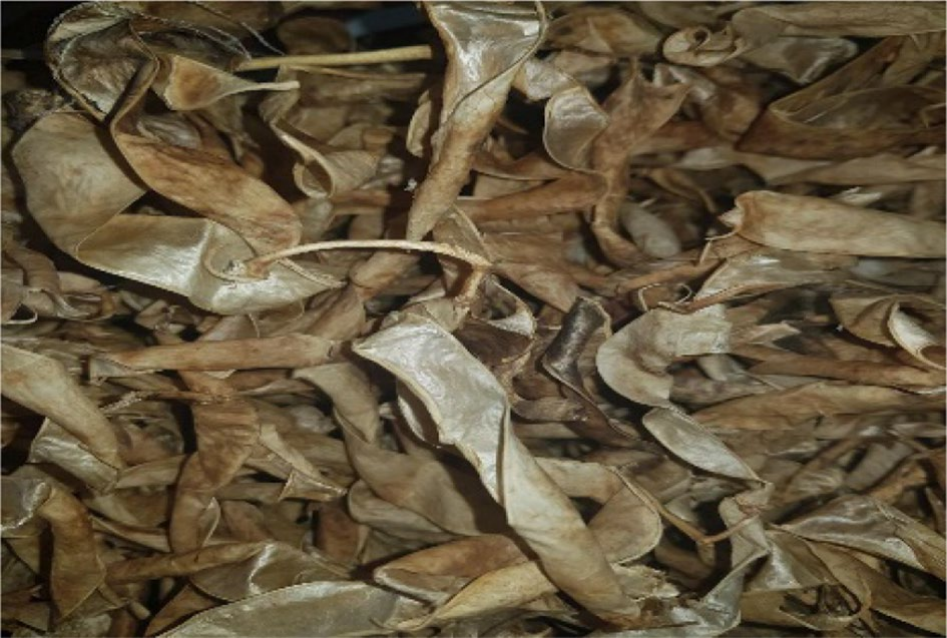
Bean bark (Bb).

The bean peel (BP), collected in the Kabylia region (Algeria) (Fig. 1), was dried, crushed, sieved to a granulometry smaller than 1 mm; thoroughly washed with distilled water to remove impurities and salts until pH ~ 7 was reached, and then dried at 50 °C (48 h).

The moisture content (H), is determined by treating the adsorbent for 24 h at 105 °C according to the standardized method NF-T 60-305 (AFNOR 1982) (Table 1):

1 $${\text{H }}\left( \% \right) = \frac{{{\text{m}}_{0} - {\text{m}}_{1} }}{{{\text{m}}_{0} }},$$

where m_o_ is the biosorbent mass before drying (g) and m_1_ the mass after drying (g). The content of the mineral matter of the biosorbent is calculated from the AFNOR-NF 04-208 method after calcination at 600 °C for 2 h:

2 $${\text{C }}\left( \% \right) \, = \frac{{m_{2} }}{{m_{1} }}.$$

**Table 1:** Characterization of Bean peel (BP) biosorbent.

Settings	Bean peel (BP)
Granulometry	≤ 1 mm
Humidity (%)	3
Bb rat (%)	4
Dry mater (%)	96
pH_ZPC_	4.8
Specific surface (m^2^/g)	5.769

The volatile matter (VM) content is given by:

3 $${\text{MV }}\left( \% \right) \, = \frac{{m_{1} - m_{2} }}{{m_{1} }}.$$

The point of zero charge (pHpzc), the pH for which the surface charge of the biosorbent is zero, was obtained from the method described elsewhere^30^. 0.05 g of biosorbent was poured in 50 mL of NaCl solutions (0.01 M) under agitation at 25 °C; the pH (2; 4; 6, 8, 10) was adjusted with NaOH and HCl (0.1 M) solutions, and the final pH was measured after 24 h.

**Figure 2:**
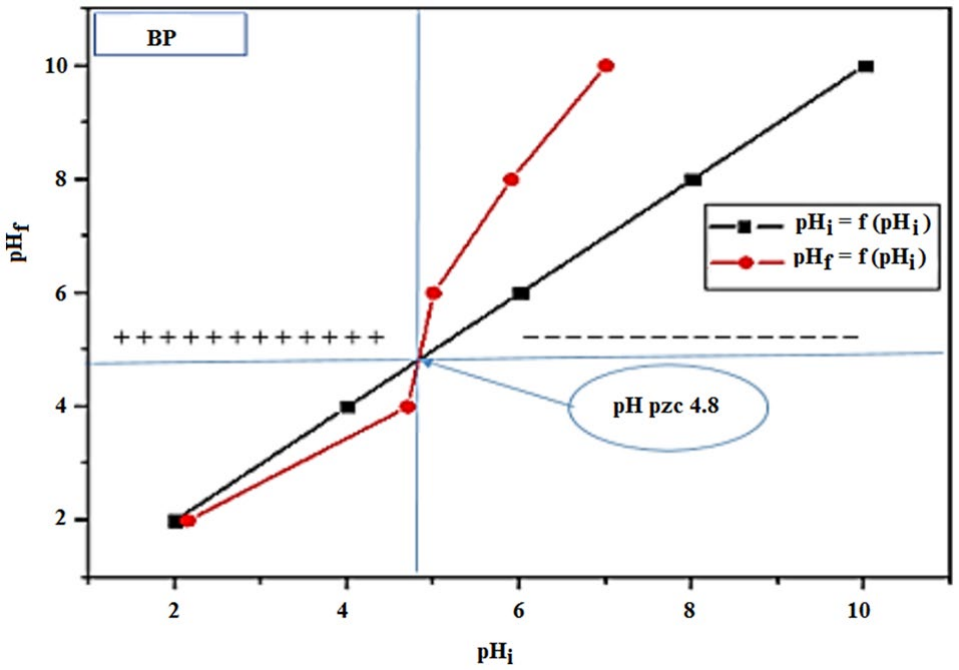
Determination of pH_PZC_ for BP biomaterial.

The straight-line pHi = f (pHi) was drawn and the intersection between the curve pHf = f (pHi) is taken as pHpzc (Fig. 2).

### Adsorbate

The Cibacron Blue (CB) is an anionic dye whose characteristics are given in Table 2; it was supplied by a local textile factory with a high purity.

**Table 2:**
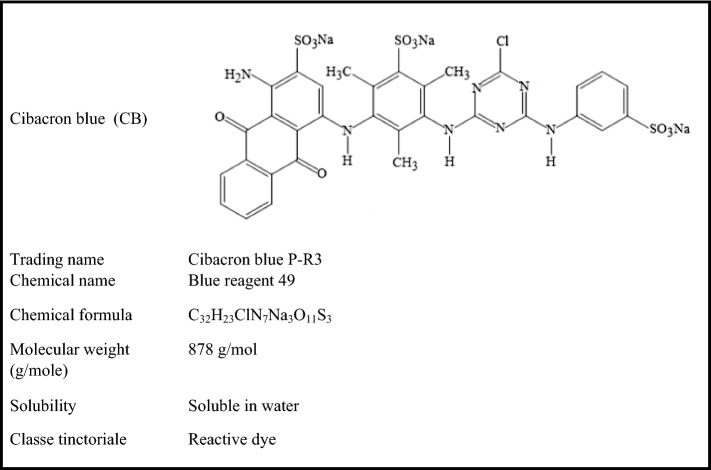
The general characteristics of Cibacron Blue.

### Methods

The Cibacron Blue (CB) concentration (10–300 mg/L) were realized by dilution of the stock solution (1000 mg/L) while the pH of the working solutions was set at ~ 2.2.

The calibration graph was plotted for several CB concentrations (3–40 mg/L) with a high correlation coefficient (R^2^ = 0.9994). The absorbance of the Cibacron Blue (CB) solutions was measured using a UV–visible spectrophotometer, UV–Vis spectrophotometer, 1601 PC-Shimadzu computer-controlled at λ_max_ = 625 nm. Distilled water was used as reference in all tests. The Cibacron Blue (CB) sorption was studied in a static tank, according to the extrinsic parameters, biosorbent mass, adsorption time, pH and initial CB concentration. The uptake yield R (%) and biosorption capacity q_e_ (mg/g) were obtained using Eqs. (4) and (5) respectively:

4 $${\text{R}}\left( \% \right) \, = \frac{{{\text{C}}_{0} - {\text{C}}_{{\text{e}}} }}{{{\text{C}}_{0} }} \times 100 ,$$

5 $${\text{q}}_{{\text{e}}} = \left( {\frac{{{\text{C}}_{0} - {\text{C}}_{{\text{e}}} }}{{\text{m}}}} \right) \times {\text{V }}{.}$$

C_i_ and C_e_ are the initial and equilibrium CB concentrations (mg/L) respectively, V the volume of the BC solution (L) and m the bean peel (g). To determine the optimal mass of the raw biomaterial, the adsorptions tests, were performed in a thermostated bath under static conditions using a back-and-forth agitator. The CB solutions (25 mg/L) and the volume (25 mL), to which different quantities of adsorbent were added (0.01, 0.09; 0.1; 0.15 and 0.20 g), maintained under stirring (2 h, 250 rpm), and decanted for 24 h. The solution was taken with a syringe without a filter for the UV–visible analysis. The adsorption capacity, was calculated according to Eq. (5). The kinetics investigations include the study of how the experimental conditions can influence the adsorption rate and bring information on the reaction mechanism, transition states and the elaboration of mathematical models that suitably describe the adsorption^31^. The biosorption was realized at optimal mass (0–120 min); the sampling took place in three times intervals (2–10 min), (15–60 min) and (70–120 min). The adsorption Kinetics is an important characteristic in the study of adsorption mechanisms. Six models, were used for this purpose: First ordre pseudo, Second ordre pseudo, Elovich's, Bangham, intraparticle diffusion and Boyd kinetic^32–36^; their equations are shown in the Table 4. To describe the ash seed adsorption mechanism onto an anionic textile dye (Cibacron blue), seven adsorption isotherm models were tested in the present study: Langmuir, Freundlich, Elovich, Temkin, Dubinin, Javanovich and BET^37–40^. Their equations, are shown in the Table 5.

The adsorption isotherm is employed to compare the uptake capacity of adsorbents. They were studied in the static mode, for the determination of maximum capacities of CB on the biosorbents. The tests were carried out on a back-and-forth agitator, and the optimal mass was determined experimentally, with the solutions (25 ml) at various concentrations (10–300 mg/L), the stirring time and decantation time were 4 and 24 h respectively.

The error analysis^41^, necessary for the evaluation of an adsorption system, is given by

6 $$RMSE = \sqrt {\frac{1}{{{\text{N}} - 2}}\mathop \sum \limits_{1}^{{\text{N}}} ({\text{q}}_{{{\text{e}},{\text{exp}}}} - {\text{q}}_{{{\text{e}},{\text{cal}}}} )^{2} } .$$

## Results and discussion

### FT-IR spectra

**Figure 3:**
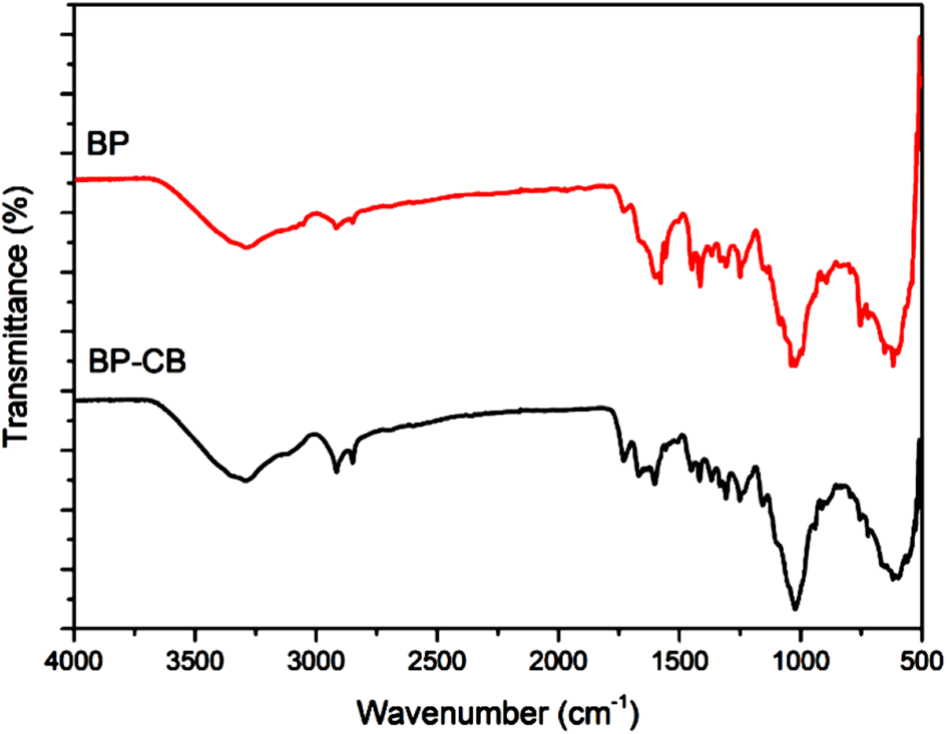
Infrared spectrum of bean bark powder before adsorption (BP) and after adsorption (BP-CB).

The FTIR results in Fig. 3 show the spectral analyses, before biosorption i.e. gross BP and after BP-CB biosorption. The FTIR spectral analysis before biosorption shows an intense band at about 3291 cm^−1^ corresponding to the stretching vibrations (–OH) of the hydroxyl groups of the cellulose and polysaccharide groups.

The observation of a peak at about 2921 cm^−1^ is attributed to asymmetric or symmetric stretching vibrations (C–H)_n_ of the methyl (–CH_3_–) and methylene (–CH_2_–) groups, as expected for hemicellulose, cellulose and lignin^42^. The 1731 cm^−1^ band associated with C=O stretching of ketones, lactones or carboxyl groups^43^. The bands detected at about 1578 cm^−1^ and 1600 cm^−1^ are attributed to the C=C double bonds in the aromatic rings of lignin. The presence of the peak at 1449 cm^−1^ is due to the vibrations of the carboxylic and lactonic C=O groups. The band observed at 1307 cm^−1^ reflects the symmetrical or asymmetrical valence vibrations of the carboxylic groups of the pectins^44^. Whereas the 1249 cm^−1^ band corresponds to the asymmetrical C–O–H bending^45^. The peak at 1022 cm^−1^ is attributed to C–O–C stretching vibrations in cellulose, lignin and hemicellulose. The weak and acute peak at 896 cm^−1^ is attributed to the vibration of the glycosidic bonds due to the presence of polysaccharides. After the treatment (BP-CB) we observe the presence of stretching vibration of the dye group C–Cl at 500–700 cm^−1^, vibration of a dye band S–O and S=O at 1022 cm^−1^ and 1250 cm^−1^. In addition, the bands at 843 cm^−1^ and 831 cm^−1^ are derived from the vibration of the primary and secondary amine groups of the dye molecules^46,47^. Thus, the FTIR spectra show the displacement of certain functional groups and other groups that appear from the CB dye fixation on the surface of the biomaterial BP.

### SEM analysis

**Figure 4:**
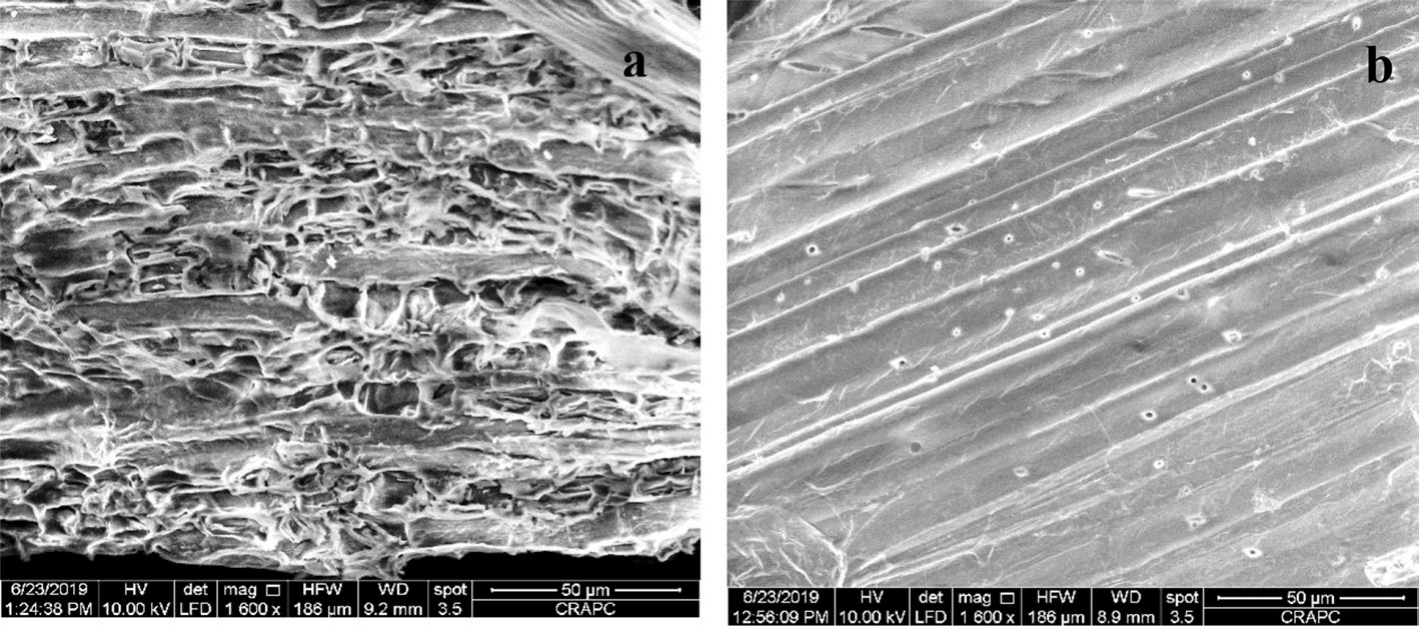
SEM images for Bean peel (BP) before adsorption (**a**) and Bean peel-dye (BP-CB) after adsorption (**b**).

The SEM analysis shows the porosity of the biomaterial with micrographs of different magnifications (Fig. 4). Before adsorption, the SEM micrograph of BP shows an average size of ~ 50 μm and presents some cavities and asperities of different sizes. The SEM images after CB biosorption by BP, the biomaterial surface has become smooth with formation of a thin layer due to dye adsorption.

### Effect of mass adsorbent

**Figure 5:**
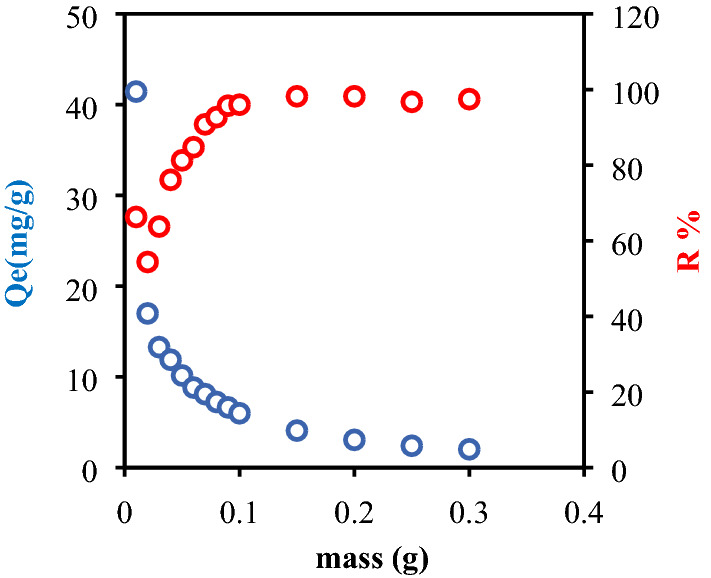
Effect of the dose of the adsorbent on the rate and quantity of adsorption dye CB by bean peel (BP) (C_0_ = 25 mg/L, V = 25 mL, T = 25 °C, t = 2 h).

The biosorption of the Cibaron Blue dye was studied in batch mode, varying the mass of the biosorbent from 0.01 to 0.20 g. The biosorption tests were done with 25 mL of CB at a concentration of 25 mg/L, at a pH of 2.2 and a temperature of 25 °C. (The experiment redone three times). The mass of the biosorbent is an important parameter in making the whole process feasible and applicable on an industrial scale^48^. Figure 5 shows that increasing the dose of biomaterial leads to a decrease in the biosorption capacity per unit mass (mg/g), due to the unsaturation of the adsorption sites. From a mathematical point of view, the biosorption capacity is inversely proportional to the dose of adsorbent. Thus, its increase causes a direct decrease in adsorption capacity while the decrease in biosorption capacity (q) is due to the superposition and aggregation of biosorption sites^49^. The CB removal efficiency over BP is 95.31% at 0.09 g / 25 mL, and increasing the adsorbent mass increases the active surface area, and the availability of adsorption sites.

### Effect of pH

**Figure 6:**
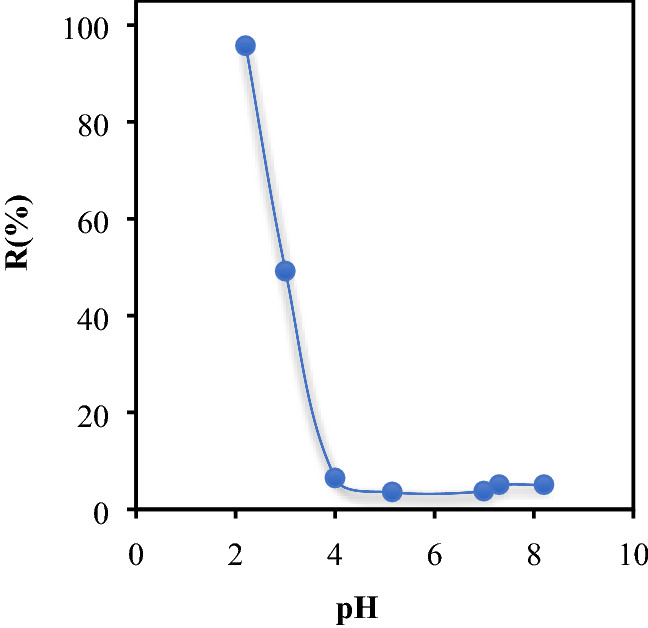
Effect of pH on the adsorption of CB by Bean peel (BP) biosorbent (m_Bb_ = 3.6 g/L, C_0_ = 25 mg/L, ω = 250 tr/min, T = 25 °C et t = 2 h).

The pH is a crucial parameter controlling the biosorption mechanism and has a great effect on the adsorbed amount and its elimination rate in the medium. It can change the surface charge of the biosorbent, the ionization degree of the adsorbate and the dissociation of functional groups of the biomaterial. The pH effect of the CB solution on the capacity of BP, was investigated for pH ≤ pHzpc. The effect of pH of CB solution on the capacity of BP was investigated for pH ≤ pHzpc. The tests were realized in a discontinuous regime using 25 mL of colored solution at a concentration of 25 mg/L and the optimal mass was determined experimentally. Figure 6 shows the yield (%) of CB as a function of pH. These results enable us to deduce that the biosorbent charge is positive for pH < pH_pzc_. The elimination of CB by BP reaches 95.73% and increases with decreasing pH since the CB dye removal at pH 4–4.8 is low due to the competition of OH^-^ ions which preclude the CB fixation on the surface in addition to the formation of intermediate bonds biosorbent/water.

Adsorbate and adsorbent also account for the regression of activity. Conversely, for pH > 4.8 (pH > pH_pzc_) the negatively charged material, induce repulsion forces. Because it is characterized by an important surface chemistry parameter, that is pH of zero charge, (pH_pzc_) defined as the pH value at which the net surface charges (external and internal) of a biosorbent are zero. From the point of view of the pH_pzc_ definition, the study was done in the range of pH < pH_PZC_, relative to our adsorbate (anionic dye), but the other domain of definition pH > pH_PZC_ is ignored. When pH of the solution is less than pH_PCZ_ = 4.8, the biosorbent surface groups will be protonated by excess of H^+^ (i.e. –COOH_2_^+^, –OH_2_^+^).

For the pH of the solution higher than pH_PCZ_ = 4.8, the biosorbent surface becomes negatively charged due to deprotonation of the oxygen-containing surface groups (i.e. –COO– and –O–), which reinforces the electrostatic repulsion forces between the functional groups of biosorbent and the SO_3_^−^ fixing functions of CB, hence the rate of elimination does not exceed 5%, which explains why there is there is no other interaction mechanism of CB biosorption^50^. The removal rate of CB dye is highly dependent on the biosorbent pH_ZPC_.

The objective of this work is to use the biomaterial alone, without chemical or physical modification or the addition of salts (effect of ionic forces), to assess its performances. On the other hand, the effect of salts depends on the biosorbents and biosorbates; it either increases the biosorption, or there will be no improvement of adsorption. Salt alone without adsorbent, can adsorb pollutants but without providing information on the true performance of biosorbents.

### Effect of initial dye concentration

Concentrations in real effluents are generally high at the exit of the plants and the study of this parameter was undertaken in the CB range (10–300 ppm). The removal efficiency of the CB as a function of the initial concentration C_o_ is given in Table 3. As expected, the elimination rate of CB decreases gradually with augmenting C_o_, from 95.3% (20 ppm) down to 35% (300 ppm). Therefore, at low CB concentrations, the elimination exceeds 90% while at higher concentrations (150–300 ppm), the maximum CB retention is 35% because of the saturation of available sorption sites.

**Table 3:** Effect of initial dye Cibacron blue P-R3 (CB) concentration on the removal efficienty [m (Bb) = 3.6 g/L; pH = 2.2; ω = 250 tr/min; T = 25 °C; t = 2 h].

Ci (mg/L)	10	20	25	30	40	50	70	100	150	200	250	300
R %	94.11	95.33	96.00	91.56	85.67	78.67	68.89	66.55	53.44	44.32	35.82	35.12

The phenomenon of hydrophobicity is an interaction or attractive force between non-polar surfaces that appears in aqueous media^51^. hydrophobic energies are between 5 and 10 kJ/mol^52^. Extensive work has been carried out to explain the hydrophobic effect, but no theory has been able to withstand the experiment. Atomic force microscopy can be used to determine two regimes for this effect. One at a long range (between 10 Å and 200 Å) and the other at a short range (< 10 Å)^51^. Water molecules can surround a molecule of a different nature without losing hydrogen bonds (short range), whereas in our long range case water molecules have to "sacrifice" hydrogen bonds, so water molecules prefer to move away from molecules with a radius greater than about ten angstroms, creating an interface similar to a liquid/vapor interface. In the case of a large cavity within the water, the radius (long range) is greater than 10 Å, the water molecules are too far apart to form stable hydrogen bonds, the cavity is therefore hydrophobic.

Accordingly, ions or molecules of solutes (CB) locally change the structure of the water, creating an electric field through the water, which polarizes the molecules in the solution. This polarization can then create a force of attraction between two surfaces, which has the effect of repelling the water, according to the Meyer et al.^51^ hypothesis.

### Kinetic aspect of biosorption

**Figure 7:**
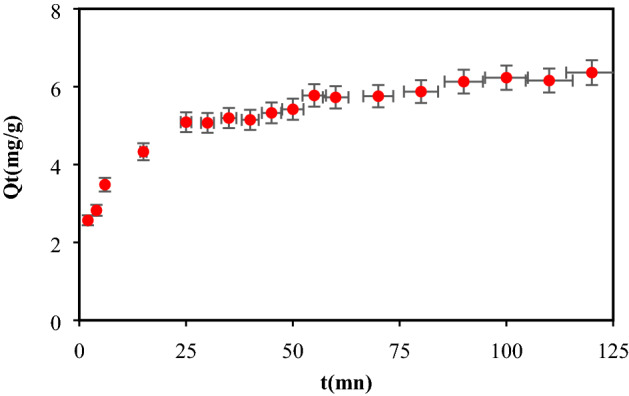
Kinetic aspect on the Biosorption of dye Cibacron blue P-R3 (BC) (m_Bb_ = 3.6 g/L, C_0_ = 25 mg/L, pH = 2.2, T = 25 °C, ω = 250 tr/min, t = 2 h).

The kinetic study enables to highlight the CB biosorption mechanism (Fig. 7). Two segments are observed; we can observe a rapid biosorption at the beginning (3–25 min) due to the availability of opening active sites on the biosorbent surface.

For the second segment, only the maximum amount adsorbed at equilibrium, is observed at 5.72 mg/g for BP while the rest of the unadsorbed quantity is due to the saturation of sites. To better understand the kinetics and mechanisms of biosorption, various models, are reported in the literature. The pseudo-first-order, pseudo-second-order, Elovich model, intraparticle diffusion model, Boyd model and Bangham models; were tested to describe the BC dye adsorption on our biomaterial.

According to the results obtained (Table 4), the experimental values and those calculated for pseudo-first-order model have shown that the adsorbed quantity has no correlation despite the high correlation coefficients.

**Table 4:** Parameters of the kinetic models studied for Cibacron blue biosorption on Bean peel (BP) biosorben.

Kinetic models	Parameters	CB (C_i_ = 25 mg/L)
	Q_exp_ (mg/g)	5.72
Pseudo-first-order $log\left( {q_{e} - q_{t} } \right) = log\left( {q_{e} } \right) - \frac{{K_{1} t}}{2,303}$	K_1_ (1/mn)	0.0216
	Q_e_ (mg/g)	3.3319
	R^2^	0.9674
	RMSE	0.3151
Pseudo-second-order $\frac{{\text{t}}}{{{\text{q}}_{{\text{t}}} }} = \frac{1}{{{\text{K}}_{2} {\text{q}}_{{\text{e}}}^{2} }} + \frac{1}{{{\text{q}}_{{\text{e}}} }}t$	K_2_ (g/(mg mn)	0.0199
	Q_e_ (mg/g)	6.5659
	R^2^	0.9959
	RMSE	0.4030
Intra-particle diffusion $q_{t} = K_{id} t^{0,5 } + C$	K_id1_ (mg/g/mn^0,5^)	0.7131
	C_1_ (mg/g)	1.5576
	R^2^	0.9866
	K_id2_ (mg/g/mn^0,5^)	0.2390
	C_2_ (mg/g)	3.7730
	R^2^	0.9500
	RMSE	0.1065
Bangham $log\left[ {log\left( {\frac{{C_{0} }}{{C_{0} - q_{t} *m}}} \right)} \right] = log\left( {\frac{{K_{\beta } *m}}{2,303*V}} \right) + \alpha logt$	α < 1	0.225
	K_β_ (L/g.ml)	8.4334
	R^2^	0.9750
	RMSE	5.5227
Elovich $q_{t} = \frac{1}{\beta }ln\left( {\alpha \beta } \right) + \frac{1}{\beta }lnt$	α (mg/g/mn)	6.0014
	β (g/mg)	1.0438
	R^2^	0.9884
	RMSE	0.1260
Boyd $B_{t} = - 0,4977 - ln\left( {1 - \frac{{q_{e} }}{{q_{t} }}} \right)$	D_i_ (Cm^2^/s)	5.50 × 10^–6^
	R^2^	0.9608

It appears that for the fixation of CB on the biomaterial BP, the adsorbed quantity obtained experimentally is 5.72 mg/g, which agrees with that obtained by the second-order model with a coefficient R^2^ of 0.9959 and Q_e (cal)_ of 6.56 mg/g.

This suggests that the adsorption process can be a chemisorption in most cases, but the physiorption is not excluded. To confirm the hypothesis, it is necessary to examine the thermodynamic parameters that are essential in the biosorption of CB^53^. In addition, the Elovich model applies well to the biosorption of BC on the biomaterials (R^2^ = 0.9884), which is close to unity.

When applying the Weber and Morris model, the adjusted curve does not go through the origin, and this indicates that the intra-particle diffusion, does not limit phase, which describes the kinetic process of CB dye binding.

Therefore, this process occurs in two different stages: a diffusion through the outer film and the boundary layer of the surface of the adsorbent followed by the intra-particle diffusion; so we can suggest that these two steps may be involved in the adsorption mechanism. Boyd’s model predicts a slow step in the process of CB fixation. The obtained line does not go through the origin, indicating thereby that the external diffusion is the decisive step in CB biosorption, with a diffusion coefficient Di equal to 5.50 × 10^–5^ cm^2^/s. This value is the range (10^–6^–10^–8^ cm^2^/s) and the kinetic is controlled mainly by external diffusion^54^. Finally, the Bangham model shows also a good linear regression (R^2^ = 0.9750). Both the surface and diffusion of the pore are important at different times in the adsorption process^55^. The best fit of the kinetic model is Elovich, according to the calculation of RMSE.

**Figure 8:**
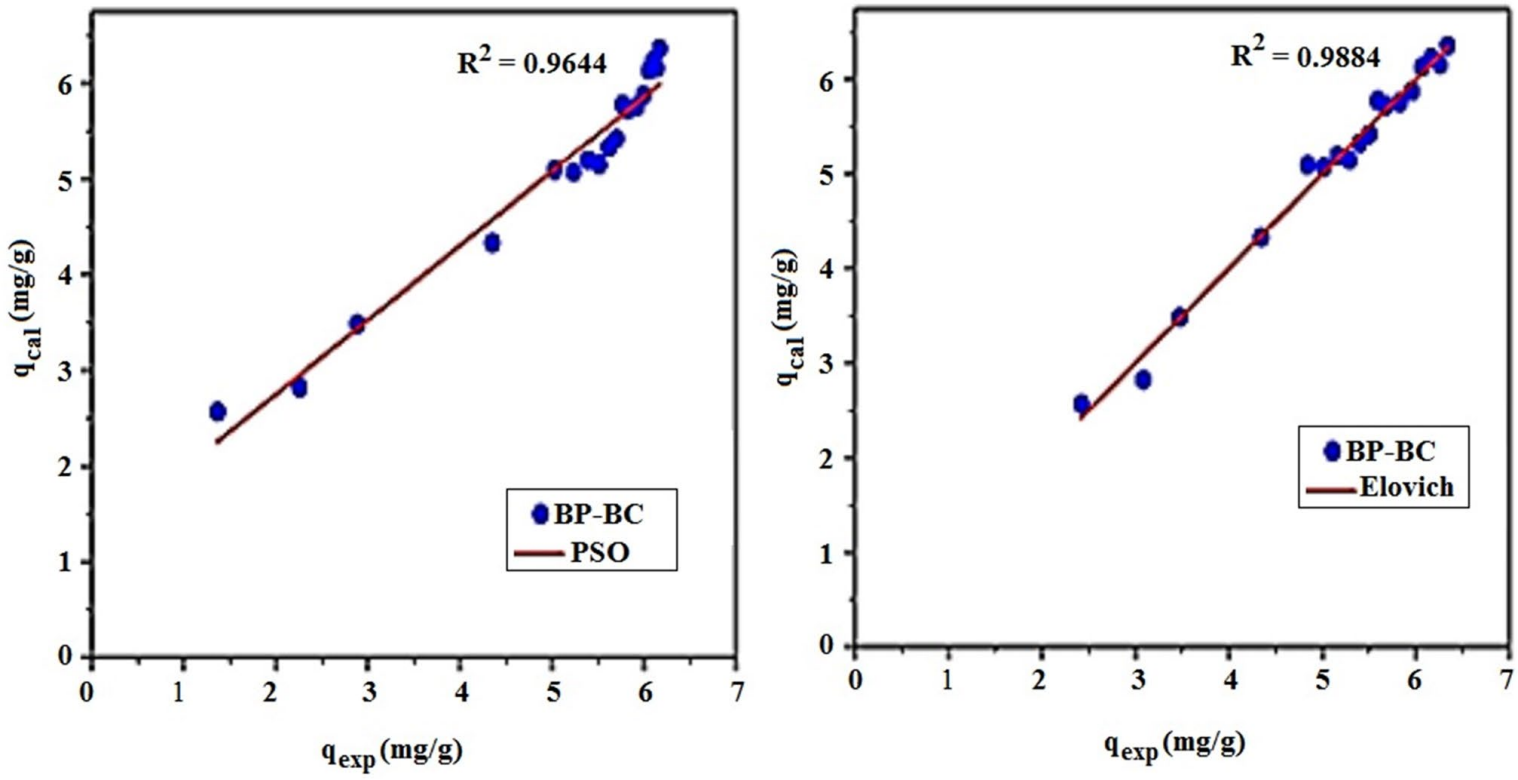
Influences data calculated by kinetic models on experimental data.

The verification by the tracing method the data calculated by the kinetic models on the experimental data that is—a—i.e. Q(cal) = f(Q(exp)), showed that the best fit, is the Elovich model (chemisorption model) with R^2^ = 0.9884. On the other hand the correlation coefficient for PSO is R^2^ = 0.9644 see Fig. 8.

### Sorption experiment

**Figure 9:**
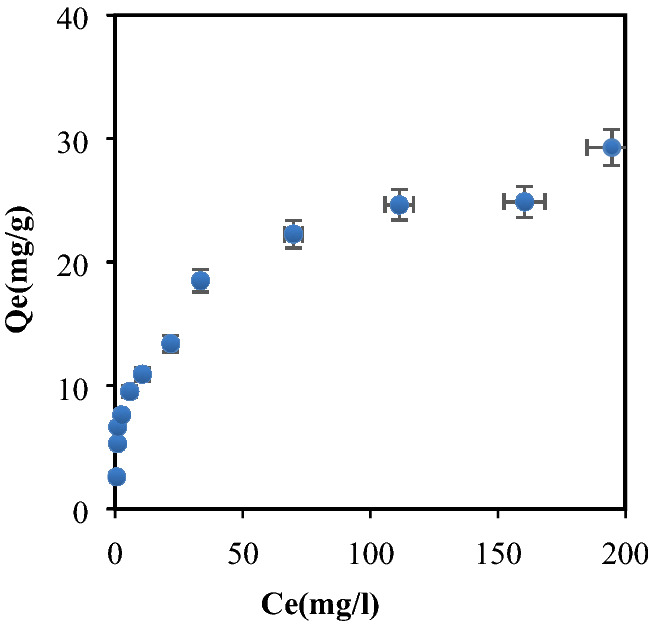
Isothermal adsorption of CB on bean peel (BP) (m_(Bb)_ = 3.6 g/L; C_i_ = 10–300 ppm; pH = 2.2; ω = 250 tr/mn; T = 25 °C; t = 4 h).

The adsorption isotherms play an important role in the determination of the maximum capacities and in the identification of the type of adsorption by the representation q_e_ = f (C_e_). Our experimental data were adjusted to the models of Freundlich, Langmuir, Temkin, Elovich, BET, Dubinin-Radushkevich and Jovanovic; their validity was evaluated through RMSE (Table 5). For BP-CB, the model that perfectly describes the adsorption process is that of Langmuir with a high coefficient R^2^ = 0.9822, a low RMSE = 2.9852 and a separation factor (R_L_), which determines the affinity between the adsorbent and the adsorbate, can be calculated by application: R_L_ = 1/(1 + KL) × C_0_; lying between 0 and 1^37^. For C_o_ = 25 mg/L, R_L_ = 0.33; while for Co = 300 mg/L, R_L_ = 0.04 ; indicating a favorable biosorption. The maximum biosorption capacity (q_max_) was estimated to be 28.49 mg/g for the Langmuir II isotherm. The maximum capacity is consistent with the experimental capacity (Fig. 9).

**Table 5:** Parameters of isothermal models studies for Cibacron blue biosorption on Bean peel (BP) biosorbent linearization.

Type	Cibacron blue (CB)
Langmuir II	$\frac{{C_{e} }}{{q_{e} }} = \frac{1}{{q_{m} }} \times \frac{1}{{C_{e} }} + \frac{1}{{q_{m} K_{L} }}$
q_m_ (mg/g)	28.490
K_L_ (L/mg)	0.081
R^2^	0.9822
RMSE	2.9852
Freundlich	$lnq_{e} = \frac{1}{n}lnC_{e} + lnk_{F}$
K_f_ (L/mg)	4.965
1/n_f_	0.3418
R^2^	0.9414
RMSE	1.5152
Elovich	$ln\frac{{q_{e} }}{{C_{e} }} = lnK_{E} q_{m} - \frac{{q_{e} }}{{q_{m} }}$
q_m_ (mg/g)	6.662
K_E_ (L/g)	0.320
R^2^	0.9241
RMSE	7.7458
Temkin	$q_{e} = \frac{RT}{{\Delta Q}}lnK_{T} + \frac{RT}{{\Delta Q}}lnC_{e}$
ΔQ(KJ/mol)	0.598
K_T_(L/g)	2.726
R^2^	0.948
RMSE	2.1358
Dubinin-R	$lnq_{e} = lnq_{m} - \beta \varepsilon^{2}$
q_m_ (mg/g)	16.771
β (mol^2^/KJ^2^)	3E−06
R^2^	0.6828
RMSE	7.1266
Jovanovic	$lnq_{e} = q_{m} - K_{J} C_{e}$
q_m_ (mg/g)	7.6453
K_J_ (L/g)	− 0.0085
R^2^	0.6131
RMSE	5.6121
BET	$\frac{{C_{e} }}{{q_{e} \left( {C_{s} - C_{e} } \right)}} = \frac{1}{{q_{m} C_{BET} }} + \frac{{C_{BET} - 1}}{{q_{m} C_{BET} }} \times \frac{{C_{e} }}{{C_{s} }}$
q_m_ (mg/g)	44.642
C_BET_	15.163
R^2^	0.9162
RMSE	2.5262

The models of Temkin, BET, Elovich and Freundlich were also appropriately applied with R2 and RMSE, respectively, as follows: R^2^ = 0.948 and RMSE = 2.1358, R^2^ = 0.9162 and RMSE = 2.5267, R^2^ = 0, 9241 and RMSE = 7.7458, R^2^ = 0.9415 and RMSE = 1.5115. This implies that the model which describes the adsorption process well is that of Freundlich with a very low RMSE, and a constant 1/nf = 0.3418 in the range [0.1] or nf = 2.9256 in the range [1.10], which indicates a favorable adsorption^56^. In addition, the Langmuir model has a correlation coefficient R^2^ = 0.98 close to unity, which suggests that adsorption of the CB dye occurred in monolayer and multilayer^57^. The CB physisorption is confirmed by the thermodynamic study (ΔH < 0; ΔG < 0, Table 6).

**Table 6:** Thermodynamic parameters of Cibacron blue biosorption on Bean peel (BP).

T(K)	ΔH (kJ/mol)	ΔG (kJ/mol)	ΔS (J/Kmol)
298	− 32.363	− 4.887	− 92.210
303	− 32.363	− 4.426	− 92.210
308	− 32.363	− 3.965	− 92.210

### Thermodynamic studies

For thermodynamic studies**,** the standard free energy change (ΔG°), enthalpy (ΔH°) and entropy (ΔS°) were calculated to determine the nature of BC biosorbption^58,59^:

7 $$\Delta {\text{G}} = - {\text{R}} \times {\text{T}} \times \ln {\text{K}}_{{\text{C}}} { ,}$$

8 $${\text{K}}_{{\text{C}}} = \frac{{{\text{C}}_{{{\text{ads}}}} }}{{{\text{C}}_{{\text{e}}} }},$$

9 $$\Delta {\text{G}}^{^\circ } = \Delta {\text{H}}^{^\circ } - {\text{T}}\Delta {\text{S}}^{^\circ } .$$

C_ads_ is the difference between the initial concentration and the remaining CB concentration in solution. ΔH° and ΔS° were calculated from the slopes and intersections of the ln K_C_ plots against 1/T:

10 $${\text{lnk}}_{{\text{c}}} = \frac{{\Delta {\text{S}}^{^\circ } }}{{\text{R}}} - \frac{{\Delta {\text{H}}^{^\circ } }}{{\text{R}}} \times \frac{1}{{\text{T}}} .$$

**Figure 10:**
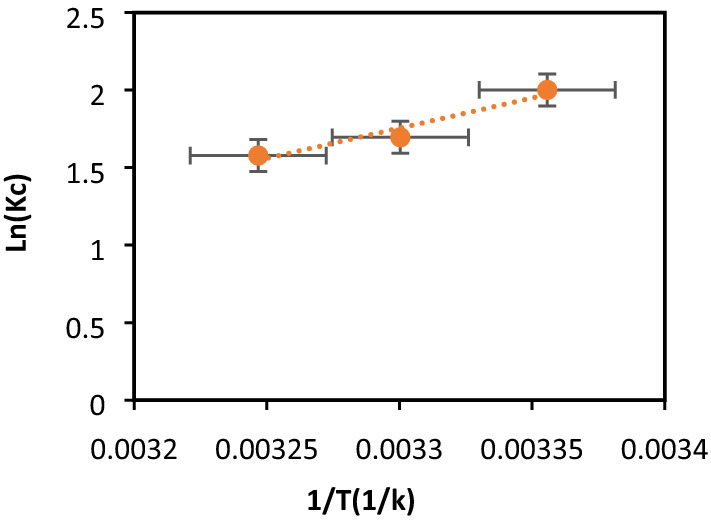
Thermodynamic studies of dye CB biosorption on bean peel (BP).

The free energy change (ΔG°), enthalpy (ΔH°) and entropy (ΔS°) were calculated to determine the nature of CB dye biosorption on native BP (Table 6, Fig. 10). ΔH° and ΔS° are deduced from the slope and the ordinate at the origin of the line lnKc = f (1/T). The negative enthalpy ΔH indicates that the CB physisorption on BP with an exothermic nature and weak attraction forces. Physisorption is a spontaneous process that makes the adsorbate-adsorbent system more stable. An adsorbate-adsorbent interaction is created, so adsorption is generally exothermic, ΔH^0^ (biosorption) < 0, promoted by a drop in temperature. Adsorption generally does not modify the biosorbent surface; the variation in biosorbent entropy is therefore negligible. The adsorbate entropy variation is negative as far as the adsorbate is structured on the biosorbent surface, ΔS° (biosorption) < 0. The negative entropy ΔS° suggests a decrease in the disorder at the interface biosorbent/solution.

The free energy ΔG° is negative and increases with rising temperature, confirming a spontaneous CB dye biosorption. In general, for the physisorption ΔG° lies between − 20 to 0 kJ/mol with Van Der Waal forces, electrostatic interactions and hydrogen bonds^60^.

Generally, several mechanisms can take place between adsorbate CB dye and biosorbent BP, including non-covalent interactions, electrostatic attraction interactions, dipole–dipole, hydrogen bonds, Van der Waals, nucleophilic interactions, π–π and n–π interactions, and whith have been suggested for the removal of inorganic and organic contaminants from the aquatic environment^61–63^. Electrostatic attraction interactions can occur between negatively charged sites on the surface of BP and anionic CB molecules in solution, when the solution pH is below pH_ZCP_, the functional groups of pectin, cellulose and lignin on the surface of BP will be ionized (i.e. –COOH_2_^+^, OH_2_^+^) and the binding functions of CB dye SO_3_^−^. Hydrogen bonding can occur between the surface hydrogens of the hydroxyl groups (H-donors) of the BP adsorbent (–OH from cellulose, lignin, pectin and tannins) and the appropriate atoms (i.e. nitrogen, oxygen and chlorine; H-acceptors) of the CB adsorbate (this phenomenon is also known as the dipole–dipole hydrogen bond^61^; and between the hydroxyl groups on the surface of BP and the aromatic rings of CB (this phenomenon is also known as the Yoshida hydrogen bond). Based on FTIR analysis, one could confirm the existence of an H-donor group during the CB biosorption process. In Fig. 2 a shift of the –OH group vibrations wave from 3291 to 3292 cm^−1^ waves is observed after CB biosorption, confirming the existence of dipole–dipole interactions and Yoshida hydrogen bonding^61,64^. Another possible adsorption mechanism is the n–π interaction, which occurs between atoms rich in long pair electrons, such as oxygen on the surface of BP and π electron cloud of CB molecules^63^. Van der Waals interactions exist between all atoms and molecules and are of low intensities (2 to 4 kJ/mol^65^. Three types of interactions can be differentiated: Keesom, Debye and London interactions, without ruling out nucleophilic interactions.

## Conclusion

The present work presents the elimination of Cibacron Blue, an anionic dye, using as biomaterial native bean peel derived from plant waste precursors available in Kabylia region (Algeria). The FTIR spectroscopy of the waste before and after biosorption, suggests that the BP uptake occurred by physisorption through Van der Waal type interactions and hydrogen bonds. Parametric optimization of physical factors yielded satisfactory results. The optimal dose for the CB biosorption were 90 mg per 25 mL; the best biosorption efficiencies are found at pH 2.2. The pseudo-second-order agrees well with the experimental data, but the Elovich model describes well the adsorption process where external diffusion is the limiting step.

Kinetic and isotherm studies show that the biosorption of CB on BP occurred in monolayer and multilayer, and that the thermodynamic parameters indicate an exothermic biosorption with a decrease in the randomness of the solid/solution interface. The free enthalpy confirms an exothermic and physical biosoprtion with weak interactions. According to the performances obtained in the present work, these precursors plant wastes are less expensive, locally available and promising alternative for the elimination of dyes in real textile effluents.

## Nomenclature

CB: Cibacron Blue
BP: Bean peel
FTIR: Fourier Transform InfraRed spectroscopy
SEM: Scanning electron microscopy
BET: Brunauer, Emmett and Teller theory
RMSE: Root-mean-square error
AFNOR: French Association for Standardization
ΔH: Enthalpy (kJ/mol)
ΔG: Change in free energy (kJ/mol)
ΔS: Entropy (J/kmol)
R: Removal (%)
C_0_: Initial concentration of CB (mg/L)
C_e_: Equilibrium concentration of CB (mg/L)
q_e_: Amount adsorbate adsorbed at the equilibrium (mg/g)
V: Volume of dye solution put in contact with the adsorbent (L)
m: Mass of adsorbent (g)
t: Time of contact (min)
q_t_: Amount of adsorbate adsorbed at time t (mg/g).
K_1_: The pseudo-first order rate constant (min^−^^1^)
K_2_: The pseudo-second order rate constant (g/mg/min).
K_id_: Intra-particle diffusion rate constant (mg/g/min)
K_β_: Canstante bangham (L/g mL)
α: The initial adsorption rate of Elovich (mg/g/min)
β: The Elovich constant for desorption in an experiment (g/mg)
K_F_: The Freundlich constant (L/mg)
K_L_: Langmuir affinity constant (L/mg)
q_m_: The maximum adsorption capacity of the adsorbent (mg/g)
n_F_: Freundlich’s constant (related to adsorption intensity)
K_E_: Elovich adsorption constant linked to the affinity of the surface sites with the adsorbate (L/mg)
K_T_: Temkin constant (L/mg)
ΔQ: Change in adsorption energy (J/mol)
β_D_: This is the Dubinin–Radushkevich constant, related to the adsorption energy
K_J_: Jovanovic constant (L/mg)
ω: Speed of stirring (tr/min)
